# The Dynamic Range Paradox: A Central Auditory Model of Intensity Change Detection

**DOI:** 10.1371/journal.pone.0057497

**Published:** 2013-02-28

**Authors:** Andrew J.R. Simpson, Joshua D. Reiss

**Affiliations:** Centre for Digital Music, Queen Mary University of London, London, United Kingdom; University of Salamanca- Institute for Neuroscience of Castille and Leon and Medical School, Spain

## Abstract

In this paper we use empirical loudness modeling to explore a perceptual sub-category of ***the dynamic range problem*** of auditory neuroscience. Humans are able to reliably report perceived intensity (loudness), and discriminate fine intensity differences, over a very large dynamic range. It is usually assumed that loudness and intensity change detection operate upon the same neural signal, and that intensity change detection may be predicted from loudness data and vice versa. However, while loudness grows as intensity is increased, improvement in intensity discrimination performance does not follow the same trend and so dynamic range estimations of the underlying neural signal from loudness data contradict estimations based on intensity just-noticeable difference (JND) data. In order to account for this apparent paradox we draw on recent advances in auditory neuroscience. We test the hypothesis that a central model, featuring central adaptation to the mean loudness level and operating on the detection of maximum central-loudness rate of change, can account for the paradoxical data. We use numerical optimization to find adaptation parameters that fit data for continuous-pedestal intensity change detection over a wide dynamic range. The optimized model is tested on a selection of equivalent pseudo-continuous intensity change detection data. We also report a supplementary experiment which confirms the modeling assumption that the detection process may be modeled as rate-of-change. Data are obtained from a listening test (*N* = 10) using linearly ramped increment-decrement envelopes applied to pseudo-continuous noise with an overall level of 33 dB SPL. Increments with half-ramp durations between 5 and 50,000 ms are used. The intensity JND is shown to increase towards long duration ramps (*p*<10^−6^). From the modeling, the following central adaptation parameters are derived; central dynamic range of 0.215 sones, 95% central normalization, and a central loudness JND constant of 5.5×10^−5^ sones per ms. Through our findings, we argue that loudness reflects peripheral neural coding, and the intensity JND reflects central neural coding.

## Introduction

Human hearing is known to function over an extremely wide dynamic range. In contrast, at a neural level the auditory system is known to have a very limited dynamic range. In auditory neuroscience, this is known as *the dynamic range problem*. In this paper we address a somewhat paradoxical sub-category of the dynamic range problem which has arisen in psychoacoustics.

Loudness (*L*) is the perceived intensity (*I*) of a sound and the just-noticeable change in intensity is called the intensity just-noticeable difference (JND). Both loudness and intensity change detection are typically assumed to operate upon the same neural signal, generated in the cochlea and transmitted on the auditory nerve. This assumption gives rise to the intuitive anticipation of a relationship between loudness and the intensity JND, such that one may be predicted from the other and vice versa. However, previous workers [1]–[3] were not able to provide a unified model due to the apparently paradoxical observation that loudness growth, beyond a certain level, is not reflected in improvement in intensity discrimination performance [3], [4]; the large dynamic range implied by loudness data is in contradiction of the relatively small dynamic range implied by intensity JND data.

The work of Hellman and Hellman [1], [2] and Allen and Neely [3] resulted in the theoretical construct of the loudness JND, which represents the just-noticeable change in loudness that corresponds to the intensity JND, and the assumption that a reciprocal relationship between loudness and loudness change detection should exist. Focusing on the intensity *discrimination* paradigm, Hellman and Hellman [1] predicted loudness functions for pure tones from intensity JND data, following the suggestion of McGill and Goldberg [5], [6] that the loudness JND is the square root of loudness (Δ*L_jnd_* = *L* ^0.5^). Allen and Neely [3] tested this for tones and noise using equivalent loudness and intensity JND (Δ*I_jnd_*) data as follows:
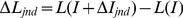
(1)


Using the loudness function of Fletcher and Munson [7] and the equivalent intensity discrimination data of Riesz [8], Allen and Neely showed (via Eq. 1) that the square root exponent of Hellman and Hellman [1] required modification above 20 dB sensation level (SL) and introduced a ‘saturation of internal noise’ to account for the modification. This showed that loudness and loudness change detection may not be modeled reciprocally and thus, their paradox was defined.

**Figure 1:**
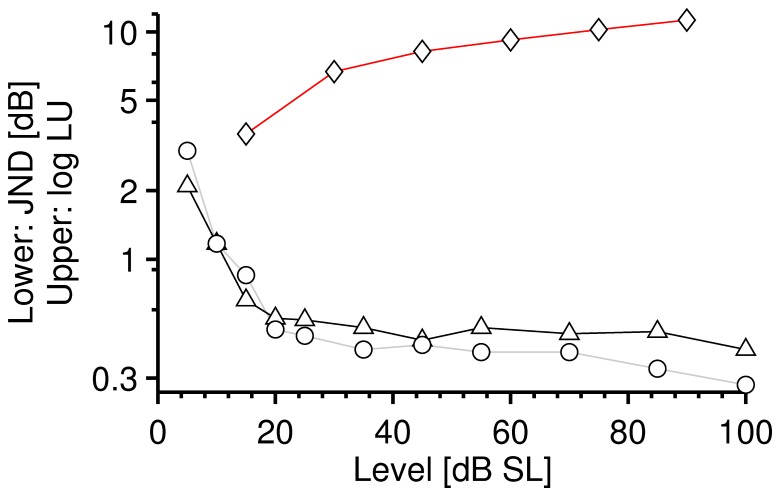
Loudness versus intensity JND. Miller’s averaged data for loudness (diamonds) and the intensity JND (circles/triangles) for broadband noise for two individual listeners, as a function of sound level (SL). Loudness data (diamonds), presented in log loudness units (LU), are taken from Neely and Allen who converted them from loudness level data of Miller using the loudness function of Fletcher and Munson. Above about 20 dB SL the JND is approximately constant (i.e., Weber’s Law) but loudness increases.

To illustrate the paradox, Fig. 1 shows a comparison of Miller’s [4] wide-band noise data for the intensity JND and for loudness levels as a function of intensity. Miller’s [4] loudness level data are converted into loudness units (LU), taken from Neely and Allen [9] according to the loudness function of Fletcher and Munson [7], and plotted in *log*(LU) for comparison to the intensity JND. At medium levels and above, loudness rises while the intensity JND remains almost constant.

Recent auditory neuroscience literature appears to provide a promising solution; Dean *et al.* [10], [11], Wen *et al.* [12] and Rabinowitz *et al*. [13] have addressed the dynamic range problem in terms of adaptive neural coding. It has been demonstrated (in animals) that central neural adaptation to mean sound level acts to improve coding of sound at the most likely (mean) sound level, mitigating neural dynamic range limitations. Dean *et al.* [10] showed that input/output functions of neuronal populations in the inferior colliculus of the guinea pig are able to shift their operating points to suit the prevailing (most likely) stimulus sound pressure level. Dean *et al.* also showed that the result of such neural adaptation may be characterized as an imperfect dynamic-range normalization of the neural signal. The general parameters that define the adaptation process are the time constant (how fast the adaptation occurs), threshold (central neural dynamic range) and amount (how much adaptation occurs).

In order to resolve the paradox, in this paper we assume that central adaptation to mean sound level occurs in humans during psychoacoustic experiments [14]. We also assume that the small change that constitutes a typical intensity JND falls at the lower limit of the fixed central neural dynamic range, and that adaptation to high mean levels necessarily raises the lower limit accordingly. This adaptive raising of the lower limit effectively degrades intensity discrimination performance relative to the performance limitations imposed by the peripheral processor.

There are no physiological data available to characterize central adaptation in human listeners. Therefore, in a numerical optimization sense, the time constant, threshold and amount are effectively free parameters within an empirical model of central adaptation. The main object of this paper is to establish, by a process of optimization, working central adaptation parameter values from the empirical data available in the psychoacoustic literature.

While there are data available over a wide enough dynamic range to establish the free parameters of adaptation threshold and amount, the majority of psychoacoustic experiments on intensity discrimination do not control or report the mean sound level over the entire course of the experiment. Hence, there are no data available to establish the time constant.

To overcome this problem, we look to the continuous-pedestal (carrier) paradigm, where the reported pedestal level provides a good approximation to the long-term average level. Two such studies exist with data over a very wide dynamic range; one for tones [15] and the other for noise [4]. Both studies remain definitive, in terms of data and in terms of phenomena characterized by the data, and are ideal for our optimization problem.

The theoretical foundation for our modeling is the excitation pattern model [16]. The excitation pattern model is an empirical model of the cochlea and auditory nerve representation of a sound – hence we may classify it as a *peripheral model*. The output of this model may then be integrated in order to calculate loudness [17]. This is known as the integrated auditory nerve formulation of loudness [7], [3].

The excitation pattern loudness model [17] incorporates functionality, based on peripheral auditory physiology, which approximates the major phenomena of psychoacoustic theory (i.e., cochlear compression, spread of excitation, the auditory filter, etc). The parameters of the model are set to fit a broad range of empirical data. We take this model as input to our central model, much as the auditory nerve is peripheral to the (central) auditory cortex. We extend the peripheral excitation pattern model to include a central adaptive representation which we call a *central excitation pattern model*. This approach is similar to that of Parra and Pearlmutter [18], who proposed a central adaptive model of tinnitus and the ‘Zwicker tone’.

Since the excitation pattern model of loudness is well established, we optimize the central adaptation parameters of our central excitation model to relate the fixed parameters of the loudness model to intensity change detection. In keeping with the paradoxical data, we make the implicit assumption that loudness, and loudness change, are coded independently at a central neural level, based on common input from the auditory nerve.

In the first stage of this study we briefly review the related literature and describe an analysis of the empirical data based on simulation of the experiments that produced the data. This analysis is used to assess the scope of the problem. Next we propose a central excitation pattern model with a maximum rate-of-change detector. The free parameters of the model are optimized to fit the tone and noise intensity JND data over a wide dynamic range. The resulting optimized model is shown to perform well at predicting independent pseudo-continuous intensity JND data from the literature. In the Experiment S1 section, an experiment is reported, based on the detection of linearly ramped up-down increments in pseudo-continuous noise pedestals. This experiment shows that slowly-ramped increments are hard to detect and validates our use of a rate-of-change model. In this article we provide empirical evidence to support an argument that loudness reflects peripheral coding, and the intensity JND reflects central coding.

## Methods

We base our analysis, and subsequent modeling, on the time-varying excitation pattern loudness model of Moore *et al.* [17], [19] – which we term *peripheral*. The model has been adequately described by the authors and we do not repeat the description here except to summarize the temporal integration of the model. Glasberg and Moore’s time-varying loudness model [19] produces a time-varying excitation pattern which is integrated over short time intervals to produce ‘instantaneous loudness’. Two successive exponential temporal windows are then used to estimate short-term loudness (STL) with respect to instantaneous loudness, and long-term loudness (LTL) with respect to STL. STL is used to account for loudness of brief duration sounds of fixed intensity, and LTL is used to account for overall loudness impression of continuous amplitude modulated sounds.

Each temporal window is defined by a pair of exponential functions and time constants for ‘attack’ and ‘release’ respectively. The STL integration times are not symmetrical, the attack time is 25 ms and the release time is 50 ms, in order to account for greater forward masking than backward masking. The attack and release times for LTL are similarly asymmetrical. The attack time is 100 ms and the release time is 2 s, allowing for the persistence of loudness impression after the stimuli has ceased.

Because the present study is concerned with amplitude modulation for continuous pedestals, we apply the loudness model using the LTL integration window. While the LTL attack time was deliberately set [19] to fit data for loudness of amplitude modulated sounds, the 2 s LTL release time is merely intended to produce a lasting impression of loudness after the stimulus has ceased. Since this release time is not justified, in our modeling the LTL release time was set to 100 ms (the same as the attack time), which produced a symmetrical temporal window for LTL with respect to STL. The combination of the two temporal windows remains asymmetrical due to the asymmetry in the short-term temporal window.

### Magnitude or Envelope?

An important question is whether it is the size or envelope of the increment that determines the detection threshold. Hellman and Hellman [1], [2] and Allen and Neely [3] have defined the loudness JND in terms of magnitude of loudness change caused by the intensity increment (Eq. 1). This means that for envelope ramps which are long (slow) compared to temporal integration of loudness the intensity JND is assumed to be constant.

A single study exists which does not support this assumption. Riesz’s [8] study of the intensity JND is rarely considered, by today’s standards, to be strictly intensity discrimination. However, this study was the first to introduce evidence to suggest a rate-of-change detector process. It involved the detection of amplitude (or, envelope) modulation produced when two sine-waves, closely spaced in frequency, are summed to produce a modulating envelope and is known as the method of beats. Riesz used continuous 1 kHz signals to test the amplitude modulation (beat) detection thresholds, as a function of beat rate, and found the smallest thresholds at a rate of around 3–4 Hz. He also found that at lower and higher rates of modulation, the threshold of detection increased almost symmetrically (on a log-rate scale) about the 3–4 Hz point. This result is not predicted by Eq. 1. We conducted a supplementary experiment (see Experiment S1) to confirm the generality of Riesz’s results as a function of beat rate.

Eq. 1 provides a loudness domain subtraction between loudness values at two intensity levels, which relates the difference in intensity to the difference in loudness that is just noticeable by *discrimination*. However, for the rate-of-change detector necessary to explain the data of Riesz [8], this equation must be transformed into the time domain [20]. This transformation between the JND domains, for change over a given time frame (Δ*t*), relates change in intensity Δ*I/*Δ*t* to change in loudness Δ*L/*Δ*t*. Eq. 1 becomes:
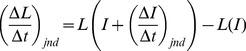
(2)


### Choice of Continuous Data

Candidate continuous-pedestal data for increment detection in noise [4] and in pure tones [15] were selected because of the large dynamic range covered in both studies (>90 dB), and because both studies remain definitive. In Miller’s [4] experiment, the increment envelope for the noise signals was instantaneous (square) and duration was 1.5 seconds. For the experiment of Viemeister and Bacon [15], tones contained 10-ms cosine-ramped increments of 200 ms duration. A full description of the stimuli of the respective studies is given in the *Description of Modeled Experimental Conditions* section.

Weber’s Law states that the ratio of the intensity JND to intensity should be constant [21]. Miller’s data showed that this was approximately true for noise signals. However, Weber’s Law does not generally hold for pure tones, as is demonstrated by the data of Viemeister and Bacon. The appearance of an ‘almost’ constant ratio for pure tones has been termed the ‘near-miss’ to Weber’s Law [5], [6]. Therefore, the two studies chosen provide a contrast, both in terms of stimuli properties (tones/noise, envelope shape, increment duration) and in terms of qualitative characterization of the data (Weber’s Law/‘near-miss’) and a compelling challenge to the intended unified model.

### Transformation of Continuous Data

Here we investigate the question of whether temporal integration of the loudness model is able to unify the two paradigms sufficiently that we can proceed to optimization of the central adaptation stage. Using the loudness model we transform *I* into *L*, Δ*I_jnd_* into Δ*L_jnd_*, and finally (Δ*I*/Δ*t*)*_jnd_* into (Δ*L*/Δ*t*)*_jnd_* for the simulated pedestals-with-increments of Miller and of Viemeister and Bacon. This analysis tells us how much need there is for central adaptation and the range in which it is necessary.

**Figure 2:**
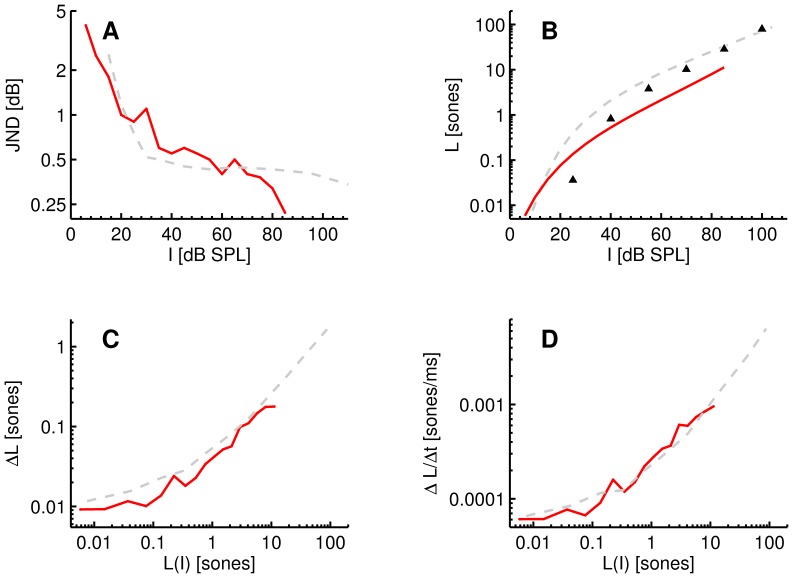
Transformation results for the noise data of Miller [dashed grey line] and the pure-tone data of Viemeister and Bacon [solid red line]. A Average intensity JND data. **B** Estimated loudness functions [*L*(*I*)] for the stimuli (pedestals). Triangles represent Millers loudness data (*I* = SL +10 dB), converted to sones (1 sone = 975 LU) from the calculated values of Neely and Allen. **C** Eq. 1: Estimated transformation of Δ*I_jnd_* [pane **A**] to Δ*L_jnd_*. **D** Eq. 2: Estimated transformation of Δ*I_jnd_* [pane **A**] to (Δ*L*/Δ*t*)*_jnd_.* The two magnitude-of-loudness-change functions in **C** are not consistent at low levels – there is an offset, but the rate-of-loudness-change functions in **D** are closer, indicating that the temporal parameters (duration, envelope) of the stimuli represented in **D** allow the stimuli to be unified. In **D**, below ∼0.25 sones the functions are approximately zero slope [i.e., (Δ*L*/Δ*t*)*_jnd_* is constant].

Fig. 2(a) shows the re-plotted intensity JND data for Miller and Viemeister and Bacon, illustrating the disparity in function shape that must be overcome within our model. Fig. 2(b) shows the loudness functions of intensity for the pedestals of the respective studies, as estimated using the loudness model. In Fig. 2(b), for comparison to the loudness model results, we also show the loudness level data of Miller [4], as converted by Neely and Allen [9] using the loudness function of Fletcher and Munson [7] (*I* = *SL* +10 dB [4]; 1 *sone* = 975 *LU*). The shape of the loudness function estimated by the loudness model is in good agreement with the loudness level data of Miller, but the loudness model predicts lower absolute thresholds than the data of Miller suggests (see *Description of Modeled Experimental Conditions* section).

Fig. 2(c) shows the respective estimated transformed data for Δ*L_jnd_*(*L*), using Eq. 1. Fig. 2(d) shows (Δ*L*/Δ*t*)*_jnd_*(*L*), estimated using Eq. 2 for Δ*t* = 1 ms. In Fig. 2(d) the two functions are much closer than the two functions of Fig. 2(c). This shows that, within the loudness model, the temporal parameters of the stimuli (envelope and duration) allow us to better unify the Δ*I_jnd_* data between the tone and noise studies in terms of (Δ*L*/Δ*t*)*_jnd_*(*L*). In other words, Eq. 1 does not take into account the envelopes of the stimuli but, using Eq. 2, the 10-ms cosine-ramped increments in tones (Viemeister and Bacon) and the instantaneous changes in noise (Miller) produce similar maximum loudness slopes for a given overall pedestal loudness.

In Fig. 2(c), we see a disagreement between the transformed data sets with regards to the smallest Δ*L_jnd_* that is detectable, by a factor of around two. This disparity would make it difficult to model using a magnitude of change model. Moore *et al.* [17] suggest an absolute threshold of 0.003 sones. Assuming that absolute threshold and masked threshold are equivalent, this is not compatible with the minimum loudness JND of around 0.01 sones shown in the function of Fig. 2(c). Therefore, it is clear that a magnitude-of-change model, with a threshold of 0.003 sones, would not explain the data.

After transforming the data further into (Δ*L*/Δ*t*)*_jnd_,* in Fig. 2(d) we see that the smallest (Δ*L*/Δ*t*)*_jnd_* is much more in agreement between the two stimuli (∼5×10^−5^ sones/ms). Thus, we confirm that our choice of decision variable [(Δ*L*/Δ*t*)*_jnd_*] is useful. The slopes of these functions, below about 0.25 sones, are relatively flat; between 0.005–0.25 sones there is a slope of around 0.00005 sones/ms but between 0.05 and 2.5 sones there is a far greater slope. These two observations conform to the two necessary conditions of constructing a central, adaptive rate-of-change model; i) that the (Δ*L*/Δ*t*)*_jnd_* functions must be close together (equivalent) and ii) that both functions must be approximately constant in the range below an equivalent loudness threshold (i.e., the two functions represent the same central dynamic range). The point where the two functions take on a marked increase in slope (∼0.25 sones) is the starting point in our search for a common threshold parameter value. During the subsequent optimization, we take the value 5.5×10^−5^ sones/ms of (Δ*L*/Δ*t*)*_jnd_* as a constant for our modeling. This might be taken to represent internal noise level.

### Central Excitation Pattern Model

**Figure 3:**
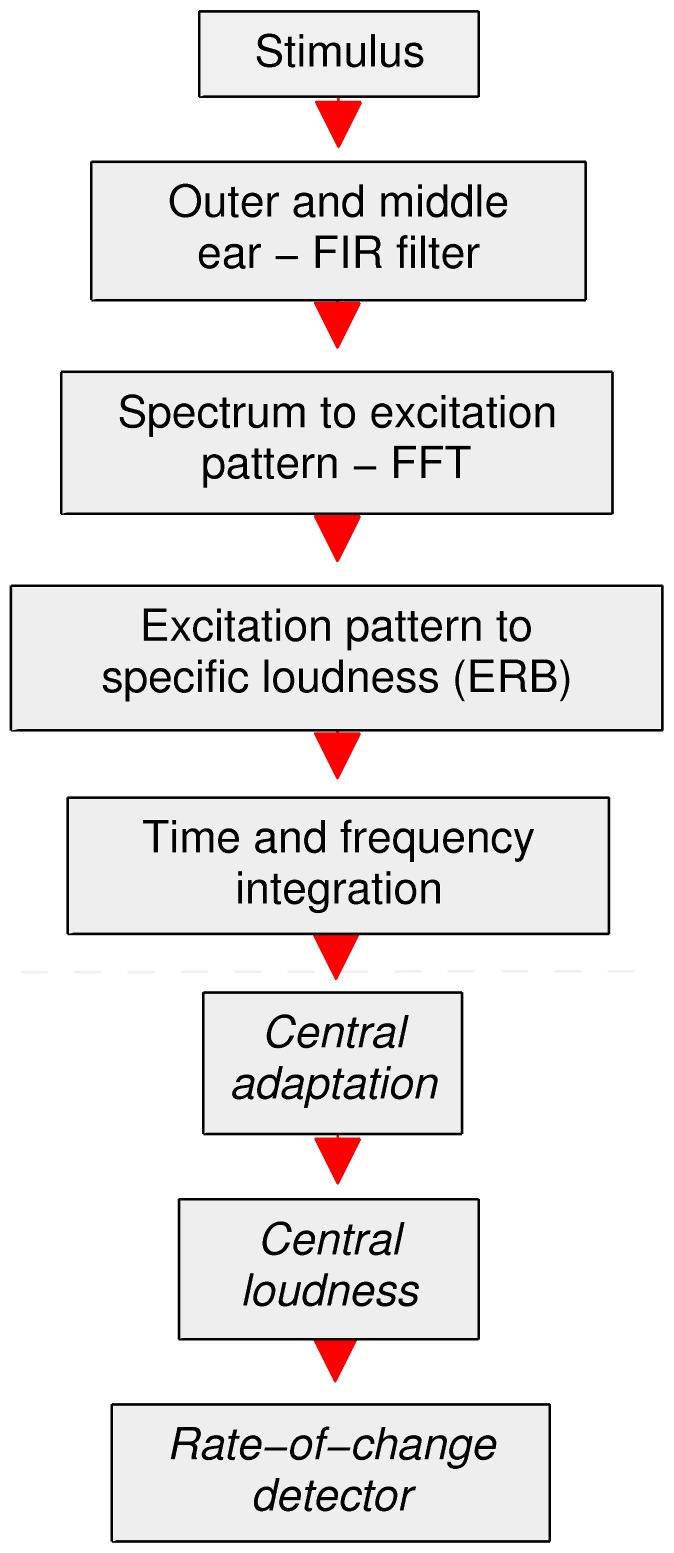
Block diagram of the central excitation pattern model and rate-of-change detector process. The area indicated as peripheral contains the loudness model of Glasberg and Moore [19] and the area indicated as central contains the proposed additions of the present study.

A general block diagram of the proposed central excitation pattern model and rate-of-change detector is given in Fig. 3. Glasberg and Moore [19] provided a loudness model that operates on the temporal waveform of a given sound to produce a time-dependent loudness function. We extend this model to produce a time-dependent central loudness contrast function which can be used to predict those changes in the intensity of a sound that may be detectable. It should be noted that our definition of central loudness (change) is purely functional/notational, in order to maintain some consistency with the previous literature regarding the loudness JND.

### Central Loudness Adaptation

Due to our confinement to the continuous pedestal paradigm, we are able to assume that mean level is approximately the same as the reported level of the pedestal. Therefore, only two free parameters are needed to define central adaptation (CA) in our model; threshold (*T_CA_*) and normalization amount (*α*). The value of *α* determines central threshold shift that results from mean peripheral loudness exceeding the central adaptation threshold (i.e., exceeds the central dynamic range). Consistent with long-term central adaptation to the prevailing sound level [10]–[13], central adaptation is implemented in the form of a partial normalization of any time-varying loudness function (*L*) which has a mean loudness (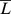) above the central adaptation threshold, *T_CA_*. Since we are concerned with continuous pedestals, mean loudness refers to a single value for tonal pedestals and an average over an arbitrarily long time frame for noise pedestals. The use of the mean loudness for adaptation threshold in continuous pedestals also provides for smoothing of instantaneous loudness changes in noise pedestals. The conditional normalization used to produce the central loudness function, *L_Cen_*, is
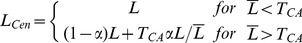
(3)


### Central Loudness JND

Unlike tonal pedestals, noise pedestals include inherent loudness changes which must be taken into account [22]–[24]. In our model we treat each noise signal as deterministic (and repeatable), or frozen [25], [26] and we base detection on the difference between the maximum value of Δ*L_Cen_* for the pedestal and the maximum value of Δ*L_Cen_* during an increment/decrement applied to that pedestal.Consistent with Eq. 2, the threshold constant is defined in sones per ms and the proposed threshold expression is
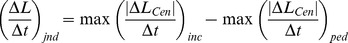
(4)where the pedestal signal is denoted (Δ*L*_Cen_/Δ*t*)*_ped_*, and the pedestal-plus-change signal is denoted (Δ*L*_Cen_/Δ*t*)*_inc_.* Thus, given a fixed (constant) value for (Δ*L*/Δ*t*)*_jnd_*, Eq. 4 may be solved by adjusting the increment size so as to affect (Δ*L*_Cen_/Δ*t*)*_inc_*.

Using a fixed value of (Δ*L*/Δ*t*)*_jnd_* extracted from Fig. 2d (5.5×10^−5^ sones/ms) a manual, iterative optimization process was conducted by using the central model to predict the value of Δ*I_jnd_* for each data point of the two studies using given parameter values of threshold *T_CA_* and *α*. Within each iteration the entire range of stimuli for both studies was simulated. For each simulation within a given iteration, Eq. 4 was evaluated numerically using the model to find Δ*I_jnd_*. The predicted value of Δ*I_jnd_* was compared to the respective data point and an error term calculated. For each iteration the average error term was calculated over the two datasets. This process was repeated, with adjustments made to the free parameters (*T_CA_* and *α*) in order to minimize the error terms until both slopes of the respective minima for each free parameter were located – i.e., until the values of *T_CA_* and *α* were optimal. The JND for the change in intensity (Δ*I_jnd_*) is expressed as
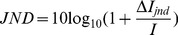
(5)


## Results and Discussion

In this section we describe the results of the optimization process and of the proposed central excitation pattern model applied to a further set of pseudo-continuous intensity JND data from the literature (see *Description of Modeled Experimental Conditions* section). For each separate simulation, within the optimization and within the simulation of the pseudo-continuous data, stimulus waveforms were produced to exactly replicate the documented conditions of the respective study. This explicitly included level and envelope.

For comparison, empirical data for intensity JND values are also presented in terms of intensity in the form of Eq. 5. Data are plotted on a logarithmic scale to allow easier determination of Weber’s Law characteristics, whilst retaining the familiar numerical scale of classical literature for the intensity JND. Goodness-of-fit measures are given, for each dataset, in the form of two-tailed Pearson correlation coefficients (*r*, *p*) and *root-mean-square* error (*e*, dB). A description of the experimental conditions for each study is given in the *Description of Modeled Experimental Conditions* section.

### Central Adaptation Parameters; Optimization Results

From the optimization, the following values were found: *T_CA_* = 0.215 sones, and *α = *0.95 (i.e., resulting in 95% normalization using Eq. 3). The *T_CA_* value of 0.215 sones (approximately 25 dB SPL in the 1 kHz pure-tone case) corresponds relatively well to the known dynamic range (approximately 35 dB) of primary auditory nerve fibers [27], [28]. The 95% normalization of the central loudness function is approximately consistent with the known sub-optimal adaptation behavior of auditory neurons [10]–[13]. In summary, the parameters found appear reasonable.

**Figure 4:**
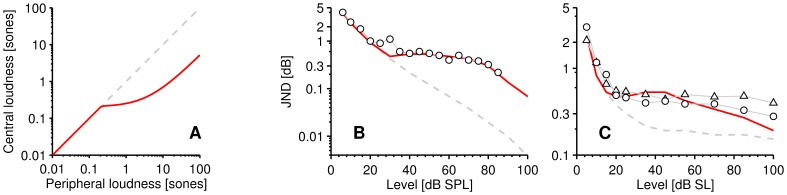
Optimization results; *peripheral versus central model*. **A** Central loudness (solid red line) for continuous pedestals, as a function of peripheral loudness (dashed grey line), illustrating the saturating effect of central adaptation (Eq. 3). **B**, **C** Comparison of estimated intensity JNDs from the peripheral and central excitation pattern rate models respectively. **B** circles: the averaged 1-kHz continuous pure-tone increment-detection data of Viemeister and Bacon and **C** is the individual (circles and triangles) continuous-noise increment-detection data of Miller.

Fig. 4(a) shows the resulting central loudness (red line) as a function of peripheral loudness (grey, dashed line), illustrating the result of the optimization and the effects of central adaptation. In order to show the effect of central adaptation on the estimated intensity JND functions, Figs. 4(b, c) show the rate-of-change predictions of the unaltered peripheral model (grey, dashed line) compared to the optimized central excitation pattern model (red line) for the data of Viemeister and Bacon (Fig. 4b) and Miller (Fig. 4c). The fit of the optimized central excitation pattern model to the data of Viemeister and Bacon is good (*r* = 0.99, *p* = 1.8×10^−13^, *e* = 0.04 dB), as is the fit to the data of Miller (*r* = 0.94, *p* = 1.4×10^−5^, *e* = 0.19 dB). The growth of loudness for both cases (tones/noise) gives a good prediction below central adaptation threshold. However, in both cases, the unaltered peripheral model results diverge strongly from those of the optimized central model above approximately 0.2 sones and the peripheral model fails to hold to the data at higher levels. As can be expected from looking at Fig. 4(b/c), the value of *T_CA_* is relatively tightly controlled by the fact that a larger value would increase the error for the data of Viemeister and Bacon (Fig. 4b) and a smaller value would increase the error for the data of Miller (Fig. 4c). The value of alpha is also relatively tightly constrained by the fact that smaller values would cause the functions to tend towards the under-estimation of the peripheral model output, and the fact that for larger values the model would tend towards Weber’s Law for the tonal data.

This modeling result is interesting because the ‘near-miss’ is often attributed to a combination of cochlear compression and spread of excitation [16], [29], where high-pass noise or high-frequency tones are used to eliminate the near-miss, and hence it is anticipated that the spread of excitation featured in the excitation pattern model should lead to a near-miss. The modeling result for the unaltered peripheral model does not produce a compelling near-miss and so it appears that the addition of central adaptation is necessary to fit the data. To repeat the statement made by Allen and Neely [3], this account of the near-miss seems different to the spread-of-excitation hypothesis.

### Results of Supplementary Experiment

**Figure 5:**
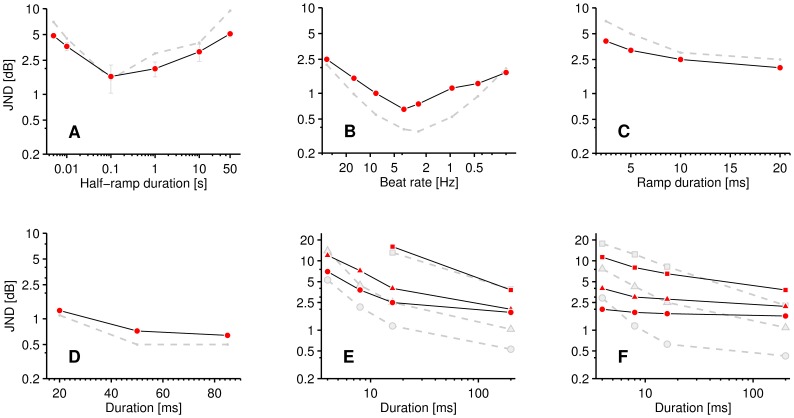
Simulation of pseudo continuous data; *miscellaneous*. Predictions of the central excitation pattern model (dashed grey line) for various data; **A** group mean thresholds of the supplementary experiment (circles); noise pedestals with up-down ramps, at half-ramp durations of 5, 10, 100, 1000, 10000 and 50000 ms and at an overall listening level of 33 dB SPL (rms). Error bars represent 95% confidence intervals. The trends shown in the data are significant (*p* = 9.55×10^−8^, *Friedman Rank Sum Test*). **B** Just-noticeable difference for envelope modulation of a 1 kHz tone, as a function of beat frequency, produced with the method of beats by Riesz for a listening level of 50 dB SL. **C** Just-noticeable difference for detection of symmetrical, linearly-ramped increments in 20-dB spectrum-level noise pedestals, as a function of half-ramp duration (one-sided) - averaged data of Plack *et al*. (circles). **D** Just-noticeable difference for increment detection in 477 Hz pedestals, as a function of increment duration at a peak level of 60 dB SPL - averaged data of Gallun and Hafter (circles). **E, F** JND for increment and decrement detection in 4 kHz pedestals respectively, as a function of duration at a listening level of 55 dB SPL - averaged data of Oxenham for 500 ms pedestals presented in quiet (circles), 0 dB (triangles) and 20 dB (squares) spectrum level noise.

Fig. 5a shows the results of the supplementary experiment (see Experiment S1). Group mean thresholds for the 10 listeners are given, including error bars representing 95% confidence intervals. The trends shown in the data are significant (*p* = 9.55×10^−8^, *Friedman Rank Sum Test*). The results, plotted on a logarithmic (time) scale, show symmetry about the half-ramp of 100-ms ‘best detection point’ which appears equivalent to that shown around 3–4 Hz by Riesz (Fig. 5b). Furthermore, the results confirm Riesz’s general finding that slow ramps are hard to detect. It should be noted that short-term (trace) memory [30] may play a role in the results at very long ramps (i.e., >4 seconds), in that the listener is forced to assess the intensity change within the short-term memory window (trace).

### Simulation of Pseudo-continuous Experiments

A selection of contemporary intensity JND studies were chosen to test the generality of the model in conditions where the continuity constraint held only loosely but where other parameters important to temporal integration theory were varied. We call these studies pseudo-continuous because the pedestals used would be considered continuous if they were not gated on and off. We also include our supplementary experiment (see Experiment S1). None of these studies varied (roved) the listening level within experimental runs, so the long-term average level should be reasonably close to the reported pedestal levels.

Fig. 5a shows the predictions of the model (dashed grey line) compared to the results of the supplementary experiment. Generally, the model predictions are reasonably close (*r* = 0.94, *p* = 4.8×10^−3^,*e* = 0.9) to the data. The model predicts an approximately symmetrical curve about the ‘best-detection’ rate. The large intensity JNDs at high and low rates of change and best-detection half-ramp duration of 100 ms are in good quantitative agreement. Within the model, Riesz’s paradigm and that of the supplementary experiment are shown to be equivalent.

Fig. 5b shows a comparison of the predictions of the model (dashed grey line) with the data of Riesz’s first experiment which determined beat-modulation intensity JND as a function of beat frequency for continuous ∼1 kHz pedestals. The shape of these data are similar to the experimental data of the supplementary experiment, in that it shows a log-time symmetrical non-monotonic JND as a function of beat rate, where low beat rates are as hard to detect as high beat rates. The shape and form of the function produced by the model is similar (*r* = 0.93, *p* = 1.4×10^−5^, *e* = 0.19 dB) to that of Riesz’s data, particularly in terms of a minimum JND point and symmetrical shape about the minimum. We note that Riesz’s data as a function of level, which almost hold to Weber’s Law above about 60 dB SL, do not appear consistent with other more recent data [20], [3] and so we do not attempt to model them here.

Fig. 5c shows the predictions of the model (dashed grey line) compared to the mean data of Plack *et al*. [31]. These data show the effect of duration on brief, linearly ramped increments in noise pedestals (see *Description of Modeled Experimental Conditions* section for further details). The model shows good agreement with the data (*r* = 0.92, *p* = 7.5×10^−2^, *e* = 0.84 dB) in terms of shape, but a small over-estimation is evident.

Fig. 5d shows the predictions of the model (dashed grey line) compared to the mean data of Gallun and Hafter [32]. These data describe the effect of brief linearly-ramped increments on 477 Hz pure tone pedestals (see *Description of Modeled Experimental Conditions* section) and so is the pure tone equivalent to the data of Plack *et al.* [31]. The model shows good agreement with the data (*r* = 0.99, *p* = 7.7×10^−2^, *e* = 0.1 dB).

Fig. 5(e, f) shows selected datapoints from Oxenham’s [33] data for brief increments and decrements (respectively) in pure-tones compared to the predictions of the model (dashed grey line). These data characterize the effect of duration and background (masking) noise on the pure tone intensity JND. The data show a monotonic decrease of JND with increase in duration and a parallel shift upwards in the JND for the addition of masking noise. In our central excitation pattern modeling of these data, we treat the sum of masking noise and tonal pedestal as a single signal and we look for a threshold increase in the maximum loudness slope caused by the increment in the tonal pedestal component. Generally, the model provides reasonable, if not ideal, qualitative and quantitative account of the data (*r* = 0.89, *p* = 2.6×10^−8^, *e* = 0.19). For the signals presented in noise, central adaptation provides for an increase of the JND consistent with the data.

Table 1 provides a summary of the goodness of fit measures described above and for the overall fit to the whole data set (*r* = 0.91, *p*<1×10^−16^, *e* = 0.09 dB). Outside of the error margins discussed in the *Error Margins* section, some error in the modeling of the pseudo-continuous data may be explained in terms of assumption of the continuous-levels approximation. It may be that the central adaptation contribution is excessive in these cases.

**Table 1:** Goodness of fit measures for the central model.

	*r*	*P*	*e*
Viemeister & Bacon, 1988	0.99	1.8×10^−13^	0.04
Miller, 1947	0.94	1.4×10^−5^	0.19
Oxenham, 1997	0.89	2.6×10^−8^	0.5
Riesz, 1928	0.93	4.8×10^−4^	0.15
Present study	0.94	4.8×10^−3^	0.9
Plack *et al.*, 2006	0.99	1.1×10^−2^	0.84
Gallun & Hafter, 2006	0.99	7.7×10^−2^	0.1
**Overall**	**0.91**	**<1**×10^−16^	**0.09**

Pearson correlation coefficients (*r*, *p*) and *rms* error (*e*) for central excitation pattern rate modeling results compared with the data.

### Summary

The main objective of this study was to establish parameters of a central adaptive model able to relate loudness to the intensity JND. The fit of the model is good, even in the case of pseudo-continuous data, and the adaptation parameters obtained are plausible with regards to the neuroscience literature.

Our supplementary experiment has shown that large intensity JNDs are obtained at very low rates of intensity change, confirming the generality of Riesz’s findings.

In the context of our modeling, we have shown that the spread of excitation explanation alone is not sufficient to produce a near-miss. Central adaptation has been used to simultaneously explain data featuring approximate examples of Weber’s Law and the near-miss, and to explain the effects of masking noise on increment and decrement detection.

In 1997, with impressive foresight, Allen and Neely suggested that the locus of their paradox might be the central nervous system. We have made explicit the argument that loudness reflects peripheral neural coding, and the intensity JND reflects central neural coding.

## Supplementary Experiment

The following experiment was designed to replicate the rate-of-change-detection paradigm of Riesz [8], within the more controlled conditions of linearly ramped increments in noise pedestals, and to confirm the generality of his rate findings. The use of linear ramps in broadband noise removes possible confounds, relating to unwanted detection cues of the beat-detection paradigm employed by Riesz.

### Ethics Statement

Participants were voluntary, unpaid and gave informed verbal consent before the experiment. Participants were free to withdraw at any point. Tests were run on an ad-hoc basis. Written consent was not deemed necessary due to the low (safe) sound pressure levels employed in the test but the consenting volunteers were documented. The experimental protocol (including consent) was approved by the ethics committee of Queen Mary University of London.

### Stimuli

All stimuli were generated digitally at 24 bit resolution. A pair of Beyerdynamic DT100 isolating headphones were used to present the stimulus to the subjects, which was played back directly from a computer at a sampling rate of 44,100 Hz. Presentation was diotic (same in both ears). The pedestal was a broadband (0-20 kHz) Gaussian noise, presented at an overall level of 33 dB SPL (rms). In the target interval, symmetrical, linearly-ramped envelopes with half-ramp durations of between 5 and 50,000 ms were added to the noise pedestals. Half-ramp durations of [5, 10, 100, 1000, 10000, 50000] ms were used. The increment consisted of a linear increment ramp immediately followed by a linear decrement ramp of equal duration. The increments were located in the temporal center of the target pedestal. For half-ramp durations of 1 second or below, pedestals were of 4 seconds. For half-ramp durations of 10 seconds, the pedestal was of 24 seconds. For half-ramp durations of 50 seconds the pedestal was of 104 seconds. Both target and reference intervals were gated with 10-ms raised-cosine ramps.

### Procedure

An adaptive three-down one-up, two-interval forced-choice (2IFC) procedure was employed which estimates the 79.4% correct identification [34]. Each trial consisted of two observation intervals, one of which was selected at random to contain the target increment. The inter-stimulus interval was 3 seconds. The level of the increment was defined as the maximum difference (in dB) between the pedestal and the target. The starting value was 20 dB. The initial step size was 5 dB for the first 4 reversals and was subsequently halved. A reversal was defined as an increase in increment size following a decrease, or vice-versa. Three consecutive correct identifications of a ramp resulted in a reduction in size of the increment and one incorrect answer resulted in an increase. After 12 reversals, threshold was taken as the arithmetic mean of the last 10 reversals.

After each trial, subjects were provided with correct/incorrect feedback on their responses. Trials were undertaken in blocks lasting no longer than 20 minutes. Due to the large number of relatively long duration trials necessary, blocks were often interrupted with a break period of 15 minutes, after which the block continued until either the next rest period or completion. For the longest half-ramp duration (50 s) such breaks were occasionally taken in the course of a single threshold determination. On two occasions, within a block, the break was extended overnight and the block was continued on the following day. Prior to the test, each subject was given a brief demonstration to familiarize themselves with the interface and procedure and was allowed a single practice run.

### Listeners

Ten unpaid volunteer subjects served as listeners in the experiments. Seven male subjects and three female subjects took part. The mean age of the subjects was 29 (min: 20, max: 36, standard deviation: 5.9). All reported normal hearing and some reported limited previous experience of participating in listening tests. All participants were naïve about the purpose of the test.

## Description of Modeled Experimental Conditions

For direct comparison with the results of Viemeister and Bacon [15], the model was used to obtain detection thresholds for increments of 200 ms in continuous 1 kHz tones over the intensity range from threshold to 85 dB SPL. The increments were gated with 10-ms raised-cosine ramps.

Miller [4] measured increment detection thresholds for two subjects using continuous, wide-band noise signals. The noise signals were specified as having power spectrum of ±5 dB from 150 to 7,000 Hz and were incremented for 1.5 sec. duration at intervals of 4.5 sec. Since Miller did not specify the spectrum outside of this range, in our modeling a band pass filter was used to reduce the energy outside of this range by 12 dB per octave. We assume that the increment envelope is square (instantaneous). Best fit to the data was found where SL was converted to SPL to be consistent with the threshold predicted by the (peripheral) loudness model (SPL  =  SL + 4 dB).

For comparison to the results of Oxenham [33], we used the model to obtain intensity JND thresholds as a function of increment and decrement duration at 55 dB SPL at durations between 4 and 200 ms. Thresholds were obtained both in quiet and in wide-band noise of 0 and 20 dB spectrum level. Increments and decrements in 4 kHz pure-tone pedestals of 500 ms were gated using raised-cosine ramps of 2 ms.

For comparison to the results of Plack *et al.* [31], the model was used to obtain thresholds for detection of brief symmetrically-ramped increments in a 20 dB spectrum-level broadband (0 - 20 kHz) noise pedestal. The ramps were linear and of durations between 2.5 and 20 ms. Increments were centrally located within the pedestal.

To test the model against the results of Gallun and Hafter [32], we employed 477 Hz pure tone pedestals and obtained thresholds for detection of brief symmetrical increments of durations between 10 and 85 ms, gated with 10-ms cosine ramps. Pedestals of 1000 ms were used and the increments were centrally located within the pedestal.

## Error Margins

There are several potential sources of error or confusion in the recreation and modeling of the experimental conditions of the studies reviewed in this paper. First, since much of the data were originally presented in terms of SL, the question of thresholds is important. Riesz [8], for example, did not obtain absolute thresholds for his subjects but took them from an earlier work by Fletcher and Wegel [35]. Fletcher and Wegel did not describe the method or statistical calculation by which they obtained their thresholds. In any case, the thresholds are sufficiently different to those obtained with modern experimental methods and equipment that some margin must be allowed to account for this. Furthermore, Miller [4] obtained absolute thresholds for his noise stimulus but did not specify the procedure by which he obtained the absolute thresholds.

Second, there is significant variation in statistical level used to define intensity JND threshold. Miller, for example, defined the threshold according to a 50% correct location on the psychometric function, whereas Viemeister and Bacon defined the threshold at the 70.7% correct point. For our supplementary experiment we define threshold at the 79.4% correct point. The model, which is based on loudness data from modern studies [17] is likely to provide error in the estimation of intensity JND values for earlier studies.

Third, the data of Miller [4] were taken with noise stimulus that is only defined as having a spectrum of +-5dB in the range of 150 Hz to 7,000 Hz. Although the +-5 dB appears reasonable for Gaussian noise, this description does not allow any reasonable assumption to be made about the spectrum of noise outside of the bandwidth specified. Further, Miller did not specify the spectrum of the noise after it had been passed through the filter of the headphone receiver. Generally, the data of the studies reviewed here were obtained with various headphone receivers and other apparatus whose influence is not known.

Fourth, the experimental population size involved in the studies reviewed is highly limited; 2 subjects for Miller, 3 subjects for Viemeister and Bacon, 4 subjects for Oxenham and 3 subjects for Riesz.

Since the intensity JND as a function of listening level is known to be a steep function at low levels, the question of absolute thresholds for a given listener or for a population is critical. Where modeling error is shown in offset but not in slope (i.e., there is an offset in the SPL axis) it is possible that variance in individual thresholds is the source of the error. This is particularly likely in light of the small population sizes described above.

### Limitations

The loudness model used here features relatively complex functionality; the transfer function of the outer and middle ear filter is relatively discontinuous, the auditory filters change shape (asymmetrically) with level and many aspects of the nonlinear input/output function are frequency dependent. Our results are therefore somewhat dependent on this model. However, alternate peripheral models should, in principle, produce similar results as far as they show an equivalent (or better) fit to loudness data.
